# The Relationship between Sympatric Defended Species Depends upon Predators' Discriminatory Behaviour

**DOI:** 10.1371/journal.pone.0044895

**Published:** 2012-09-10

**Authors:** Christina G. Halpin, John Skelhorn, Candy Rowe

**Affiliations:** 1 Centre for Behaviour and Evolution, Newcastle University, Newcastle upon Tyne, United Kingdom; 2 Centre for Research in Animal Behaviour, University of Exeter, Exeter, United Kingdom; University of Sussex, United Kingdom

## Abstract

Toxic prey species living in the same environment have long been thought to mutually benefit from having the same warning signal by sharing the education of naïve predators. In contrast, ‘saturation theory’ predicts that predators are physiologically limited by the amount of toxin that they can eat in a given time period. Therefore, sympatric species that contain the same toxin should mutually benefit from reduced predation even when they are visually distinct, reducing the benefits to visual mimicry. For the first time, we found that mutualism can occur between unequally defended prey that are visually distinct, although the benefits to each prey type depends on the predators' abilities and/or motivation to visually discriminate between them. Furthermore, we found that this variability in predatory behaviour had a significant impact on the benefits of mimicry for unequally defended prey. Our results demonstrate that variability in the foraging decisions of predators can have a significant effect on the benefits of shared toxicity and visual mimicry between sympatric species, and highlights the need to consider how predators exert selection pressures on models and mimics over their entire lifetimes.

## Introduction

Aposematic prey often defend themselves with toxins and advertise their toxicity to potential predators using conspicuous warning signals [1]. The widely held view is that aposematism is a defensive strategy aimed at naïve predators, with warning coloration evoking neophobia and dietary conservatism [2]–[5], and being easier to learn to associate with toxicity compared to cryptic coloration [6]. However, predators, both in the wild and in the laboratory, continue to eat toxic prey even when they have learned that they contain toxin, i.e. when they are ‘educated’ [7]–[12]. This is because aposematic prey contain nutrients as well as toxins, and educated predators make informed decisions based on the benefits of eating nutrients relative to the costs of eating toxins [8], [10], [13]. Knowing how educated predators make foraging decisions based on the nutrient and toxin content of prey is important since predators are long-lived compared to their prey, and are known to remember what they have learned about prey for long periods [14], [15]. It is therefore somewhat surprising that although this trade-off was initially acknowledged and discussed more than 100 years ago [1], [16], we still know very little about how educated predators make decisions. This gap in our knowledge means that we cannot fully understand the role of predator cognition in the evolution of aposematism and mimicry.

One key question is how defence strategies of sympatric species interact with one another, and specifically how the presence of one toxic species in the environment affects the survival of another [17]–[21]. Turner and Speed (2001) proposed that toxic prey that are visually distinct but share the same toxin should mutually benefit from reduced predation when they occur together, compared to when they occur alone. Their ‘saturation theory’ is based upon the idea that the number of toxic prey that an educated predator can eat is constrained by its ability to detoxify the toxin, and that two toxic species would saturate a predator's detoxification system more than either single species alone. Prey that contain the same toxin but are visually distinct could therefore be viewed as being ‘toxic mutualists’, because both species should benefit by the predator being limited to eating a fixed amount of toxin [19]. Assemblages of sympatric insect species that sequester toxins from the same host-plants do exist in nature (e.g. [22]), but we do not know if prey species sharing the same toxin do saturate predators' detoxification pathways and are indeed toxin mutualists. The only way to test this theory is to use an experimental system where the amount of toxin that a predator has eaten is known [19], and where predators have had time to learn about the toxicity of different prey and make informed decisions about what to eat.

Understanding educated predators' foraging decisions on sympatric toxic prey that are visually distinct is vital if we want to measure the benefits of Müllerian mimicry, where toxic species share the same warning signal [23]. There has been theoretical debate concerning the evolutionary dynamics of Müllerian mimicry when mimics contain different amounts of toxin [24]–[28]. Traditionally, Müllerian mimics have been thought to mutually benefit from a shared warning signal through enhanced predator aversion learning [20], [23]. However, if educated predators include toxic prey in their diets according to the amount of toxin that they contain [13], [16], [25], then the less defended mimic could potentially have a parasitic relationship with the more defended model [25]. Empirical tests of the dynamics of unequally defended mimics have been equivocal, showing various degrees of support for the theory [29]–[33]. However, these experiments have either focussed on how naïve predators learn to avoid unequally defended mimics [29], [30], [32], or have not distinguished between naïve and educated predators [31]. Therefore, we do not know whether educated predators making informed decisions about ingesting toxic prey leads to a mutualistic or a parasitic relationship between unequally defended prey.

Using an established experimental system, where starlings (*Sturnus vulgaris*) are presented with undefended and defended mealworms (*Tenebrio molitor*) [8]–[10], [34], we investigated how educated avian predators make foraging decisions on defended prey that differ in their toxin content. For the first time, we tested whether the actions of educated predators generate: (1) mutualistic relationships between visually distinct unequally defended species (by eating a fixed amount of toxin); and, (2) parasitic or mutualistic relationships between unequally defended Müllerian mimics.

## Results

**Figure 1:**
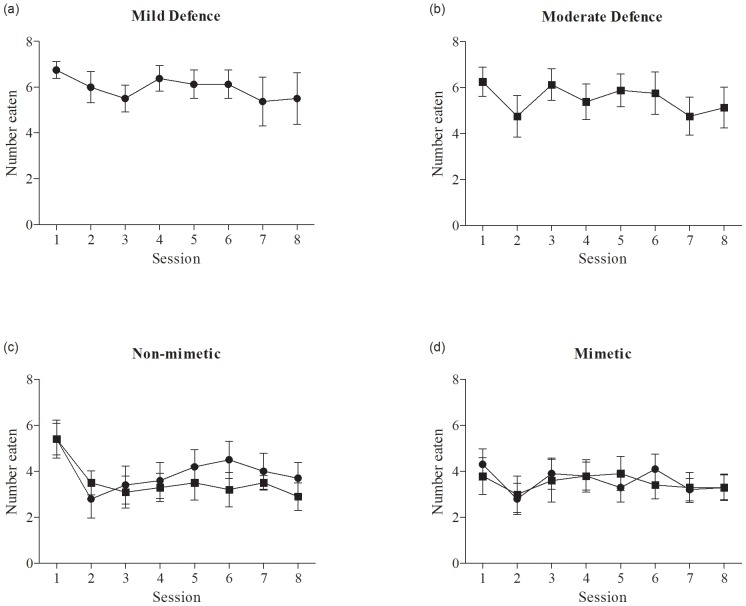
The mean numbers (+/− SE) of mildly defended (circles) and moderately defended prey (squares) eaten in each session by birds in each of the experimental groups (N = 10 for all groups).

Starlings were presented with sequences of undefended and defended mealworms on different coloured backgrounds. Defended prey were mildly and/or moderately defended depending on the experimental group (see Table 1). To measure the informed decisions and toxin intake of educated predators we first needed to establish the point at which the birds had reached a stable asymptotic attack rate on each of the defended prey types. We ran a series of repeated measures ANOVAs on the data for both mildly and moderately defended prey in all experimental groups, initially for sessions 1–8, then sessions 2–8 and then sessions 3–8. There was no significant difference in the numbers of mildly or moderately defended prey eaten in any group across sessions 3 to 8 (repeated measures ANOVA for all groups; 0.015<F_1, 9_<2.76, 0.13<P<0.90; Figure 1). We therefore concluded that the consumption of defended prey eaten in a session reached a stable asymptotic level by Session 3 for all groups, and considered the birds to be educated from Session 3 onwards. We used the data from these last 6 sessions in the subsequent analyses. Notably, the birds in all groups invariably ate almost all of the undefended prey, and there was no difference in the numbers of undefended prey eaten between the groups (F_3, 39_ = 0.56, P = 0.64).

**Table 1:** The number of each type of prey presentation made in sessions for all four experimental groups.

Group	Prey type			
	Undefended	Mildly Defended (2%)	Moderately Defended (4%)	No Prey
Mild Defence	8	8	-	8
Moderate Defence	8	-	8	8
Non-mimetic	8	8	8	-
Mimetic	8	8	8	-

### Are unequally defended non-mimics ‘toxin mutualists’?

**Figure 2:**
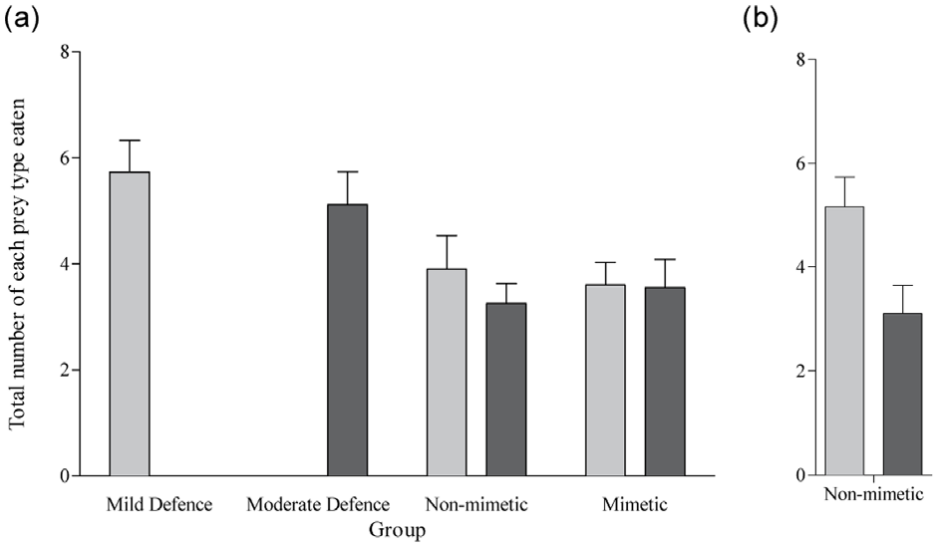
The mean (+ SE) number of mildly defended prey (light grey bars) and moderately defended prey (dark grey bars) eaten per session when birds had reached asymptote (sessions 3–8). In (a) data for all birds in all groups is included, and in (b) only data for the six discriminating birds in the Non-mimetic group is included (see text for details). Values that were significantly lower in the Non-Mimetic group compared to the Mild Defence and Moderate Defence group are marked with an asterisk. (N = 10 for all groups except the Non-Mimetic group where N = 9 in (a) and N = 6 in (b)).

We first calculated the mean number of mildly and moderately defended prey eaten per session for each bird in sessions 3 to 8. The mean number of mildly defended prey eaten per session was significantly lower when they were presented together with moderately defended prey in the Non-mimetic group compared to when they occurred alone in the Mild Defence group (independent t-test: t = 2.11, P = 0.049, df = 18; Figure 2a). Likewise, the number of moderately defended prey eaten per session was lower when they were presented together with mildly defended prey in the Non-mimetic group compared to when they occurred alone in the Moderate Defence group (t = 2.57, P = 0.019, df = 18; Figure 2a). This is the first demonstration of toxin mutualism, where visually distinct prey that share the same toxin benefit from reduced attacks from a population of educated predators.

However, it was surprising that birds in the Non-mimetic group did not visually discriminate between the two defended prey types (paired t-test: t = 1.18, P = 0.272, df = 8; see Figure 2a). Upon further inspection of the data from this group, we found that the birds clearly differed in the degree to which they discriminated between mildly and moderately defended prey. We readily labelled them as ‘discriminators’ and ‘non-discriminators’ using data from the experimental and simultaneous choice sessions. To qualify as a discriminator, a bird needed to fulfil two criteria: (i) to eat a higher proportion of mildly defended than moderately defended prey in Sessions 3 to 8; and, (ii) to eat more mildly defended prey than moderately defended prey in the simultaneous choice session. Only six birds fulfilled both criteria (see Table 2), which we will refer to as being discriminators, with the four remaining birds being non-discriminators. To statistically establish this behavioural dichotomy, we compared the discriminatory performance of discriminators and non-discriminators using the data presented in Table 2. We found that our discriminating birds ate a significantly higher proportion of mildly defended prey in sessions 3–8 (t-test: t = 5.2, P = 0.001, df = 8), and in the simultaneous choice trials (t = −3.28, P = 0.0011, df = 8) compared to non-discriminators. The difference in discrimination behaviour could have been due to the birds adopting different foraging strategies in this particular scenario, or they may have had different learning capabilities or levels of motivation. For 8 of these 10 birds in the Non-mimetic group (5 discriminators and 3 non-discriminators), we had both measurements of tarsus length and mass at the start of the experiment, from which we could calculate a condition index (mass/tarsus length). Intriguingly, we found that discriminators were significantly heavier than non-discriminators (mean condition index (±S.E.)  = 2.57±0.07 and 2.16±0.18 respectively, independent t-test: t = 2.478, P = 0.048, df = 7), suggesting that energetic state may well be a determinant in whether predators learn to discriminate between unequally defended prey.

**Table 2:** The proportion of eaten defended prey that were mildly defended in Sessions 3–8 and in the simultaneous choice session.

Bird	The proportion of defended prey eaten that were mildly defended, in Sessions 3–8	Proportion of defended prey eaten that were mildly defended in the simultaneous choice session
21*	0.62	0.86
32	0.33	0.5
36	0.36	0.25
40*	0.67	0.94
51	0.36	0.25
61	0.41	0.75
64*	0.52	0.67
65*	0.81	0.90
73*	0.59	0.78
77*	0.59	0.67

Values over 0.5 show a preference for mildly over moderately defended prey. Birds marked with an asterisk (*) were labelled as discriminating birds.

Regardless of the exact mechanism, because of the clear dichotomy in the birds' behaviour in the Non-mimetic group, we considered it important to re-analyse our data relating to toxin mutualism using only the six birds that had learned to discriminate. We did this in order to investigate what we might have found if all the birds had discriminated, and if the expression of discrimination behaviour by a population of predators could change the evolutionary dynamics between unequally defended prey. We no longer detected a significant difference in the mean number of mildly defended prey eaten per session when they were presented together with moderately defended prey in the Non-mimetic group compared to when they occurred alone in the Mild Defence group (independent t-test: t = 0.629, P = 0.539, df = 14, Figure 2b). However, the mean number of moderately defended prey eaten per session was still significantly lower when they were presented together with mildly defended prey in the Non-mimetic group compared to when they occurred alone in the Moderate Defence group (independent t-test: t = 2.198, P = 0.045, df = 14, Figure 2b). Therefore, for unequally defended, visually distinct prey, the benefits of toxin mutualism may depend upon whether or not predators learn to discriminate between the two types.

**Figure 3:**
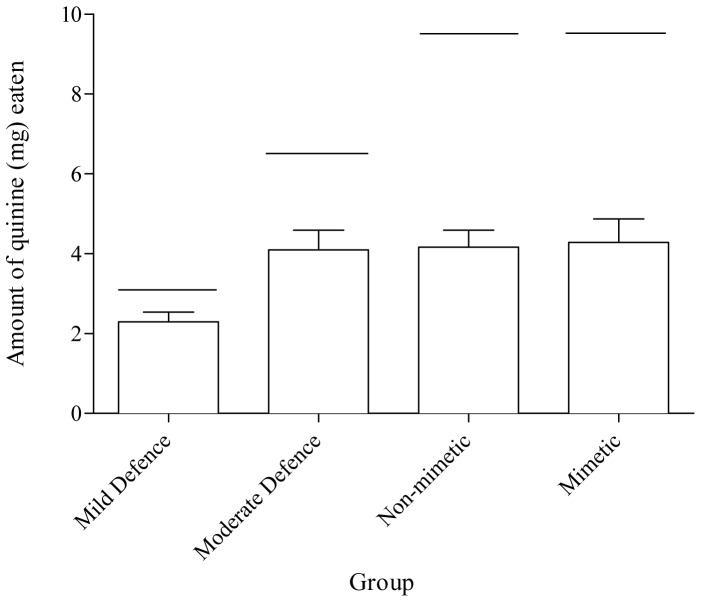
The mean (+ SE) amount of quinine (mg) eaten per session in Sessions 3–8 in each of the experimental groups. Horizontal lines on the graph indicate the maximum amount of quinine available within a session for each group. The asterisk denotes a significantly lower ingestion of quinine in the Mild Defence group compared to the other groups. (N = 10 for all groups except the Non-Mimetic group where N = 9).

Finally, to fully test Turner & Speed's (2001) saturation theory, we considered whether or not birds from our four groups ate the same amount of toxin per session. We found that the mean amount of toxin eaten per session was significantly different among our four groups when we included all the birds (ANOVA: F_3,38_ = 4.26, P = 0.011; see Figure 3) and when we included only the discriminating birds in the Non-Mimetic group (ANOVA: F_3,35_ = 4.45, P = 0.010). Post-hoc tests revealed that while birds in the Moderate Defence, the Non-Mimetic and the Mimetic groups ate the same amount of quinine (post-hoc Tukey HSD: P>0.05 for all comparisons), birds in the Mild Defence group ate significantly less quinine than birds in the three other groups (post-hoc Tukey HSD: Mild Defence – Moderate Defence, P = 0.044; Mild Defence – Non-Mimetic, P = 0.031; Mild Defence – Mimetic, P = 0.022; see Figure 3). Although this might have been expected because birds in the Mild Defence group had less toxin available to them in each trial, all groups in fact ate less toxin than the maximum presented in a trial (one-sample t-tests for all groups; 9.00>t>3.78, 0.004>P≥0.000; see Figure 3). Therefore, this does not explain why this group ate less toxin than the other three groups. This finding could not be attributed to any differences in the condition of the birds as there was no significant difference in the condition indices between groups (ANOVA: F_3, 32_ = 0.666, P = 0.579).

### Do mildly defended prey increase or decrease the mortality of moderately defended prey when they are mimetic?

To test whether visual mimicry of the mildly defended prey was either beneficial or costly to the moderately defended prey, we compared the mean number of each defended prey type eaten in a session between the Non-mimetic and Mimetic groups. Using all 20 birds from these two groups, we found no significant difference in the numbers of either defended prey type eaten (mildly defended prey: t = 0.394, P = 0.70, df = 18; moderately defended prey: t = 0.457, P = 0.65, df = 18; see Figure 2a). Therefore, surprisingly, we found that visual mimicry conferred no costs or benefits to either prey type.

However, we were also interested in whether or not there would be benefits or costs to mimicry if all our birds had become discriminators in the Non-mimetic group. We therefore repeated this analysis, now comparing the data for just the six discriminating birds with that of all the birds in the Mimetic group. We found that there was still no difference in the number of moderately defended prey eaten between Non-mimetic and the Mimetic groups (t = 0.653, P = 0.60, df = 14; Fig. 2b), but more mildly defended prey were eaten in the Non-mimetic group compared to the Mimetic group (t = 2.191, P = 0.046, df = 14; Figure 2b). Therefore, whilst we could not detect any cost or benefit to moderately defended prey through being associated with a mildly defended mimic, mildly defended prey did benefit from visual mimicry once we considered only birds that had learned to discriminate between the two prey types in the Non-mimetic group.

## Discussion

Our results show that educated predators can select for both toxin mutualism and visual mimicry between unequally toxic prey. Intriguingly, however, the degree to which birds discriminated between the unequally defended prey when they were visually dissimilar determined the relative costs and benefits for the mildly defended prey. We also found mixed evidence in support of Turner & Speed's saturation theory. We discuss each of these findings in turn.

### Are unequally defended non-mimics ‘toxin mutualists’?

Initially, when we considered the numbers of defended prey eaten using the data from all our birds, we found that both mildly and moderately defended prey benefitted from reduced mortality when they occurred together compared to when they occurred alone when they were visually distinct. These findings provide support for the idea of toxin mutualism [19]. However, when we considered only those birds in the Non-mimetic group that were actively discriminating between the unequally defended prey, the mildly defended prey no longer benefitted from the presence of the moderately defended prey, whilst the moderately defended prey did continue to benefit from the presence of the mildly defended non-mimic. Discrimination by predators is therefore a key process determining whether or not sympatric defended species containing the same toxin mutually benefit.

This finding has implications for whether or not prey would be selected to share the same toxin. The degree to which predators discriminate between unequally defended non-mimics determines how beneficial sharing the same toxin is, particularly for a less defended prey type. For example, if predators visually discriminate between two unequally toxic prey species, there may not be strong selection on the less defended prey to share the same toxin as the more defended species. We should not always predict that sharing the same toxin will be mutually beneficial [19]. In addition, the fact that predators' discriminatory behaviour varies highlights the need to understand the physiological and cognitive mechanisms underlying foraging decisions on toxic prey if we are to fully understand the selection pressures acting on prey defences. In our experiment, variability in discrimination behaviour could have resulted either from differences in learning abilities [13] or in motivation to learn. It is important to know which, since if it is learning ability, we would expect all predators to become discriminating over time. However, if motivation is the key driver, it may be that some predators never discriminate, or that predators are sometimes discriminatory and other times not. Our data suggest that the non-discriminating birds were in poorer condition than the discriminating birds, perhaps suggesting that feeding motivation is important [13]. This supports theoretical predictions that predators should only discriminate among defended prey when it pays them to do so [28]. We therefore expect that predators' decisions to discriminate (or not) would fluctuate over time according to their nutritional needs, and that the selective benefits of toxin mutualism is dynamic over time. Knowing this becomes even more important when we consider the benefits of visual mimicry (see below).

We also tested whether toxin saturation could be the mechanism underlying toxin mutualism. The toxin saturation theory predicts that a predator is constrained by the amount of toxin it can eat in a given time, and once this is reached the predator must stop consuming any prey containing that toxin [19]. Although three groups ate similar amounts of quinine in a session, birds that were given only mildly defended prey ingested significantly less quinine than the other groups. Although this was expected since they had less quinine available to them, they also ingested less than the maximum amount of quinine available in a session. This clearly shows that, contrary to the predictions of the toxin saturation theory, birds will not necessarily continue to ingest toxic prey until they reach a detoxification limit. If this were the case, we would expect the birds in the Mild Defence group to have eaten all of their defended prey. The reasons why they didn't are not clear, but our analysis on condition indices confirmed that it was not due to any differences in the condition of the birds in our experimental groups. However, the fact that their experimental sessions contained the least amount of quinine may have resulted in the birds in the Mild Defence group being in a relatively better state during the sessions compared to the other birds. This in turn may have made them less likely to eat the defended prey [10]. Whatever the reason for this difference in the amount of quinine ingested in this group compared to the others, it is clear that the behaviour of our birds cannot be fully explained by saturation.

### Do mildly defended prey increase or decrease the mortality of moderately defended prey when they are mimetic?

Theoretical models have predicted that the relationship between unequally defended prey may be either mutualistic [20] or parasitic [25], and there are empirical findings to support both sides; that models and mimics both benefit from the shared warning signal [23], [30], [35], or that a less defended mimic will be costly to a more highly defended model [32]. It is therefore perhaps surprising that we found no cost or benefit to mimicry for our moderately defended prey, irrespective of whether we considered all of the birds in the Non-mimetic group or just those which discriminated between the two defended prey types. Mildly defended prey did benefit from mimicry, but only when we considered the restricted case where birds discriminated between non-mimetic prey.

Taken together, our data show that the selection for mimicry will very much depend on whether or not predators discriminate between unequally defended prey when they are visually distinct. If visually distinct prey are already toxin mutualists, there is no additional selective advantage to mimicry from educated predators. Therefore, we might expect stronger selection for visual mimicry when prey contain different toxins and cannot be toxin mutualists, compared to when they share the same toxin (see also [19]). More importantly, if the degree of discrimination behaviour found in a predator population changes over time, either by variability in discrimination learning speed of naïve predators or in the proportions of predators that are hungry, the relative benefits to mimicry will also be dynamic. This could explain why previous experiments on the dynamics of mimicry between unequally defended prey have reported such different results [23], [30], [32], [35]. These experiments use naïve rather than educated predators, but the principle is the same: if naïve predators are quick to learn to discriminate between visually distinct defended prey, the evolutionary dynamics measured are likely to be quasi-Batesian. This is because the less defended prey will be eaten more relative to the more defended prey, and will benefit from mimicry at the expense of the more defended prey. However, if it takes predators longer to learn to discriminate between the two unequally defended prey, Müllerian mimicry is more likely to be detected. This is because the birds would be slower to associate the toxin with two signals as opposed to one [28], leading to reduced predation when both prey types look similar. Clearly, whether we are studying naïve or educated predators, we need to know how physiological and cognitive mechanisms can affect the perceived benefits to mimicry.

Our findings also demonstrate that studying how predators exert selection pressures over their entire lifetimes is going to be important to fully understand the selection pressures acting on mimicry. Although mimicry studies focus on avoidance learning in naïve predators, most predators are long-lived and make foraging decisions throughout their lifetimes based on their past experience and current needs. The selection pressures from predator populations in the wild will therefore change over time, for example, as the numbers of naïve predators increases seasonally, or changes in temperature affect foraging motivation. Therefore, we need to consider and integrate temporal changes in closer detail in order to better understand the selective pressures that are put on defended prey in the wild.

## Conclusions

We have shown for the first time that toxin mutualism can exist between unequally defended prey, although we cannot conclude that this was due to them becoming saturated with this compound. This raises interesting questions about the physiological and cognitive mechanisms underlying decisions to eat toxic prey by avian predators, since they cannot be explained by toxin regulation alone. Our findings also showed that the extent to which unequally defended prey will benefit from co-existence, whether they are visual mimics or not, will very much depend on the decisions made by the predator population. Variable and individual predatory behaviours, such as the ability or motivation to discriminate between unequally defended prey, will determine the benefits and costs of mimicry to the prey species involved. This clearly highlights that the dynamics of mimicry between unequally defended prey is likely to vary over time, and that asking whether the evolutionary dynamics of mimicry are either quasi-Batesian (parasitic) or truly Müllerian (mutually beneficial) may be the wrong question. Perhaps instead we should be asking when the dynamics are quasi-Batesian and when they are Müllerian, and how they vary over time in order to understand the benefits of mimicry.

## Methods

### Ethics Statement

The experiment was conducted under Local Ethical Committee approval (Newcastle University, ERC Project ID: 266), and in accordance with ASAB's Guidelines for the Treatment of Animals in Behavioural Research and Teaching.

### Subjects and Housing

40 (10 male, 30 female) wild-caught European starlings (*Sturnus vulgaris*) were caught under licence (Natural England 20093299) and kept in indoor free-flight aviaries. During experimental testing, subjects were housed in pairs in cages measuring 150×45×45 cm, which were enriched with perches, water baths and trays containing natural bark shavings. These home-cages were also used as experimental cages since this reduces the stress on the birds resulting from catching, handling and removal to another cage. This also reduces training times and the time spent in cages. Each cage had an opaque divider that divided the cage in half during experimental sessions. On each side of the cage there was a drawer measuring 45×75 cm, with a spring-loaded flap facing the front through which prey could be presented. Water was available at all times and food (chick crumbs, fruit and Orlux Insect Patee) was available *ad libitum*, except when birds were food deprived for 1.5 hr before a session. After the experiment the birds were returned to free-flight aviaries before release at the same site from which they were caught.

### Prey manipulations

We used mealworms (*Tenebrio molitor*) of similar length (approx. 20 mm) as prey. Mildly and moderately defended prey were mealworms injected with either 0.02 ml of a 2% or 4% quinine solution, respectively (Sigma Aldrich, Q0132–25G). Quinine has been used widely as an aversant in learning experiments (e.g. [36], [37]–[39]) and previous work has shown that it cannot be tasted when injected into mealworms in this manner [34], [40]. Instead, the birds learn to associate the post-ingestive effects of quinine with the colour cues provided (as described under *Experimental Sessions*) (e.g. [13]). Undefended prey were mealworms injected with 0.02ml of water.

### Training Sessions

A white curtain erected in front of the cage visually isolated birds during training and experimental sessions. Birds were observed via video cameras linked to television monitors, and sessions were recorded for further analysis. Subjects were randomly assigned to one of four experimental groups, which were named according to the defended prey types presented to the birds in those groups: Mild Defence Group (3 males and 7 females); Moderate Defence Group (3 males and 7 females); Non-mimetic Group (3 males and 7 females); and Mimetic Group (1 male and 9 females) (see Table 1). Birds were initially trained to eat unmanipulated mealworms out of Petri dishes. They were given a single training session on each of two consecutive days, which consisted of 24 sequential presentations of a Petri dish that either contained a mealworm, or was empty. A presentation was made every three minutes, and birds were given one minute to attack a mealworm if it was present, after which time the Petri dish was removed. Birds in the Non-mimetic and Mimetic groups were given a mealworm in every Petri dish, and birds in the Mild and Moderate Defence groups received a mealworm in 16 out of the 24 presentations, since this reflected what birds in each group received during experimental sessions (see below). The 16 mealworms and 8 ‘blanks’ were presented in a random order. After two days, all birds ate all the mealworms presented to them, confirming that satiation would not be a limiting factor to the number of prey eaten in the experimental sessions. Once they met this criterion, they began the eight experimental sessions.

### Experimental Sessions

From Day 3, birds were given one experimental session per day for eight consecutive days. In these sessions each bird was given a randomised sequence of undefended and defended prey. All birds received 8 undefended prey, but the number and the quinine content of the defended prey differed according to each experimental group. Birds in the Mild Defence group were given 8 mildly defended prey and 8 ‘blanks’ and birds in the Moderate Defence group were given 8 moderately defended prey and 8 ‘blanks’, while birds in the Non-mimetic and Mimetic groups were given 8 mildly and 8 moderately defended prey (see Table 1). Different prey types were given distinct colour signals, except in the case of the Mimetic group where the mildly and moderately defended prey shared the same signal. Colour signals were green, pink or purple coloured paper discs in the Petri dishes underneath the mealworm. Colours were counter-balanced within and between groups to control for any potential colour biases. Birds readily learn to associate the colour signals with the post-ingestive effects of the toxin (e.g. [10], [13]), and do not taste the differences in quinine concentration between prey when it is injected in this way [34], [40].

We were able to test whether the relationship between unequally defended and visually distinct prey was mutualistic by comparing the numbers of each defended prey type eaten in the Non-mimetic group compared to when they were presented singly in the Mild Defence and Moderate Defence groups. This design also enabled us to test the benefit of visual mimicry independently from the benefit of toxin mutualism, by comparing the numbers of mildly and moderately defended prey eaten in the Non-mimetic and the Mimetic groups. Crucially, since presentations were sequential and birds were given the opportunity to eat all prey, this experimental design also overcomes the problems associated with prey densities changing when prey intake is fixed (e.g. [30], [41]).

### Simultaneous choice sessions

At the end of the experimental sessions, birds in the Non-mimetic group were given an additional simultaneous choice session, where they were given the choice to eat either a mildly or a moderately defended prey. Birds in this group could learn to discriminate between mildly and moderately defended prey, on the basis of visual signals, and preferentially eat more mildly than moderately defended prey. However, if they ate equal number of each defended prey type, it could be that they either had not learned to discriminate between the two prey types or that they knew the difference between them but decided to eat them equally. This session tested whether or not birds had learned the difference between the two prey types by testing whether or on they showed a preference for the mildly defended prey when prey were presented simultaneously. Each bird was given a session of 16 paired presentations, which consisted of one mildly and one moderately defended mealworm presented singly in two Petri dishes placed approximately 10 cm apart in the cage. Each prey type had the same colour signal as in the experimental trials, and birds were given one minute to select one mealworm before both dishes were removed from the cage. Once a bird had made a choice, both dishes were removed immediately. Paired presentations were made every 3 minutes, as in the previous sessions.

## References

[1] Poulton EB (1890) The Colors of Animals., London: Trübner & Co Ltd.

[2] Rozin P (1968) Specific aversions and neophobia resulting from vitamin deficiency and poisoning in half-wild and domestic rats. J Comp Physiol Psychol 66: 82–88. 5691592 10.1037/h0025974

[3] Mitchell D (1976) Experiment on neophobia in wild and laboratory rat: A reevaluation. J Comp Physiol Psychol 90: 190–197. 1249271 10.1037/h0077196

[4] Kronenberger JP, Medioni J (1985) Food neophobia in wild and laboratory mice (*Mus musculus domesticus*). Behav Process 11: 53–59. 10.1016/0376-6357(85)90102-0 24924361

[5] Marples NM, Kelly DJ (1999) Neophobia and dietary conservatism: two distinct processes. Evol Ecol 13: 641–653.

[6] Gittleman JL, Harvey PH (1980) Why are distasteful prey not cryptic? Nature 286: 149–150.

[7] Pinheiro CEG (1996) Palatability and escaping ability in Neotropical butterflies: tests with wild kingbirds (*Tyrannus melancholicus, Tyrannidae*). Biol J Linn Soc 59: 351–356.

[8] Skelhorn J, Rowe C (2007) Predators' toxin burdens influence their strategic decisions to eat toxic prey. Curr Biol 17: 1479–1483. 17716896 10.1016/j.cub.2007.07.064

[9] Skelhorn J, Rowe C (2006) Predator avoidance learning of prey with secreted or stored defences and the evolution of insect defences. Anim Behav 72: 827–834.

[10] Barnett CA, Bateson M, Rowe C (2007) State-dependent decision making: educated predators strategically trade off the costs and benefits of consuming aposematic prey. Behav Ecol 18: 645–651.

[11] Fink LS, Brower LP (1981) Birds can overcome the cardenolide defense of Monarch butterflies in Mexico. Nature 291: 67–70.

[12] Fink LS, Brower LP, Waide RB, Spitzer PR (1983) Overwintering Monarch butterflies as food for insectivorous birds in Mexico. Biotropica 15: 151–153.

[13] Barnett CA, Skelhorn J, Bateson M, Rowe C (2012) Educated predators make strategic decisions to eat defended prey according to their toxin content. Behav Ecol 23: 418–424.

[14] Kaplan G, Rogers LJ (2001) Birds: Their habitats and skills: Griffin Press.

[15] Krebs JR, Clayton NS, Healy SD, Cristol DA, Patel SN, et al (1996) The ecology of the avian brain: Food-storing memory and the hippocampus. IBIS 138: 34–36.

[16] Marshall GAK (1908) On Diaposematism, with reference to some limitations of the Müllerian Hypothesis of Mimicry. Trans R Entomol Soc Lond 56: 93–142.

[17] Rettenmeyer CW (1970) Insect mimicry. Ann Rev Entomol 15: 43–74.

[18] Turner JR (1987) The evolutionary dynamics of batesian and muellerian mimicry: similarities and differences. Ecol Entomol 12: 81–95.

[19] Turner JRG, Speed MP (2001) How weird can mimicry get? Evol Ecol 13: 807–827.

[20] Sherratt TN (2008) The evolution of Müllerian mimicry. Naturwissenschaften 95: 681–695. 18542902 10.1007/s00114-008-0403-y PMC2443389

[21] Pinheiro C (2003) Does Mullerian mimicry work in nature? Experiments with butterflies and birds. Biotropica 35: 356–364.

[22] Tullberg BS, Leimar O, Gamberale-Stille G (2000) Did aggregation favour the initial evolution of warning coloration? A novel world revisited. Anim Behav 59: 281–287. 10675250 10.1006/anbe.1999.1302

[23] Müller F (1879) *Ituna* and *Thyridia*: a remarkable case of mimicry in butterflies. Trans Ent Soc Lond: xx–xxix.

[24] Joron M, Mallet JLB (1998) Diversity in mimicry: paradox or paradigm? TREE 13: 461–466. 21238394 10.1016/s0169-5347(98)01483-9

[25] Speed M (1993) Muellerian mimicry and the psychology of predation. Anim Behav 45: 571–580.

[26] Speed M (1999) Batesian, quasi-Batesian or Mullerian mimicry? Theory and data in mimicry research. Evol Ecol 13: 755–776.

[27] Mallet J (2001) Causes and consequences of a lack of coevolution in Müllerian mimicry. Evol Ecol 13: 777–806.

[28] Kokko H, Mappes J, Lindström L (2003) Alternative prey can change model-mimic dynamics between parasitism and mutualism. Ecol Lett 6: 1068–1076.

[29] Ihalainen E, Lindström L, Mappes J (2007) Investigating Müllerian mimicry: predator learning and variation in prey defences. J Evol Biol 20: 780–791. 17305843 10.1111/j.1420-9101.2006.01234.x

[30] Rowland HM, Ihalainen E, Lindström L, Mappes J, Speed MP (2007) Co-mimics have a mutualistic relationship despite unequal defences. Nature 448: 64–67. 17611539 10.1038/nature05899

[31] Speed MP, Alderson NJ, Hardman C, Ruxton GD (2001) Testing Mullerian mimicry: an experiment with wild birds. Proc R Soc Lond B 267: 725–731. 10.1098/rspb.2000.1063 PMC1690586 10821620

[32] Rowland HM, Mappes J, Ruxton GD, Speed MP (2010) Mimicry between unequally defended prey can be parasitic: evidence for quasi-batesian mimicry. Ecol Lett 13: 1494–1502. 20955507 10.1111/j.1461-0248.2010.01539.x

[33] Lindström L, Lyytinen A, Mappes J, Ojala K (2006) Relative importance of taste and visual appearance for predator education in Müllerian mimicry. Anim Behav 72: 323–333.

[34] Skelhorn J, Rowe C (2010) Birds learn to use distastefulness as a signal of toxicity. Proc R Soc Lond B 277: 1729–1734. 10.1098/rspb.2009.2092 PMC2871851 20129989

[35] Rowe C, Lindström L, Lyytinen A (2004) The importance of pattern similarity between Müllerian mimics in predator avoidance learning. Proc R Soc Lond B 271: 407–413. 10.1098/rspb.2003.2615 PMC1691604 15101700

[36] Alatalo RV, Mappes J (1996) Tracking the evolution of warning signals. Nature 382: 708–710.

[37] Alcock A (1970) Punishment levels and the response of Black-capped Chickadees (*Parus atricapillus*) to three kinds of artifical seeds. Anim Behav 18: 592–599.

[38] Halpin CG, Skelhorn J, Rowe C (2008) Naïve predators and selection for rare conspicuous defended prey: the initial evolution of aposematism revisited. Anim Behav 75: 771–781.

[39] Méry F, Kawecki TJ (2003) A fitness cost of learning ability in *Drosophila melanogaster* . Proc R Soc Lond B 270: 2465–2469. 10.1098/rspb.2003.2548 PMC1691529 14667336

[40] Skelhorn J, Rowe C (2009) Distastefulness as an antipredator defence strategy. Anim Behav 78: 761–766.

[41] Rowland HM, Hoogesteger T, Ruxton GD, Speed MP, Mappes J (2010) A tale of 2 signals: signal mimicry between aposematic species enhances predator avoidance learning. Behav Ecol 21: 851–860.

